# SNP markers associated with body size and pelt length in American mink (*Neovison vison*)

**DOI:** 10.1186/s12863-018-0688-6

**Published:** 2018-11-12

**Authors:** Zexi Cai, Trine Michelle Villumsen, Torben Asp, Bernt Guldbrandtsen, Goutam Sahana, Mogens Sandø Lund

**Affiliations:** 1 Center for Quantitative Genetics and Genomics, Department of Molecular Biology and Genetics, Aarhus University, 8830 Tjele, Denmark; 2 Section of Crop Genetics and Biotechnology, Department of Molecular Biology and Genetics, Aarhus University, 4200 Slagelse, Denmark

**Keywords:** American mink, GWAS, Body weight, Pelt length, Production trait

## Abstract

**Background:**

Identification of genes underlying production traits is a key aim of the mink research community. Recent availability of genomic tools have opened the possibility for faster genetic progress in mink breeding. Availability of mink genome assembly allows genome-wide association studies in mink.

**Results:**

In this study, we used genotyping-by-sequencing to obtain single nucleotide polymorphism (SNP) genotypes of 2496 mink. After multiple rounds of filtering, we retained 28,336 high quality SNPs and 2352 individuals for a genome-wide association study (GWAS). We performed the first GWAS for body weight, behavior, along with 10 traits related to fur quality in mink.

**Conclusions:**

Combining association results with existing functional information of genes and mammalian phenotype databases, we proposed *WWC3*, *MAP2K4*, *SLC7A1* and *USP22* as candidate genes for body weight and pelt length in mink.

**Electronic supplementary material:**

The online version of this article (10.1186/s12863-018-0688-6) contains supplementary material, which is available to authorized users.

## Background

American mink (*Neovison vison*) is a Mustelid species native to North America [1]. Fertility, body mass and fur quality are economically important traits for the fur industry. Scientists have made many efforts to pinpoint genes that are sources of variation in these traits. For gene mapping, a priori knowledge on the markers’ order and their locations in the genome is necessary. In 2007, Anistoroaei et al. published the first genetic map based on simple sequence repeat (SSR) markers in mink [2]. In the following years, there have been two updates of this genetic map with additional markers or using homology with dog and human [3, 4]. With this genetic map, Thirstrup et al. identified quantitative traits loci (QTL) affecting fur quality and skin length [5]. In addition, Cirera el al. found a large insertion in the *TYRP1* gene to be associated with the American Palomino phenotype [6]. Furthermore, some scientists have adopted an homology search to find genes affecting mink production traits, for example using bacterial artificial chromosome (BAC) library sequencing followed by homology search to find genes in the mink genome [7]. However, for systematic scans of the genome, identification of markers covering the whole genome is essential. Using restriction site associated DNA sequencing (RAD sequencing), Thirstrup el al. identified 380 SNPs in mink [8]. However, without information on sequences of flanking markers from a reference genome, the usability of these markers remains impeded. Recently, Cai et al. published the first draft mink genome assembly [9], which made it possible to identify SNPs across the mink genome [10] using genotyping-by-sequencing (GBS) [11]. GBS takes advantages of next-generation sequencing (NGS) technology to achieve low cost and high throughput genotyping. It uses restriction enzymes (REs) to reduce the complexity of genome followed by NGS to generate sequence tags. By choosing methylation-sensitive REs, we can avoid the genome’s repetitive regions to reduce the complexity of genome. The genome wide genetic markers obtained from GBS have become a key resource for genetics research and breeding in crops [11, 12]. Scientists adopted the same strategy in many non-model species to discover genetic markers to conduct population, genetics and genomics studies.

In mink breeding, traits related to skin size and fur quality are under strong selection, [13]. In the typical mink breeding program, the parents of production cohorts are selected based on fur quality, however the economic returns come from the dry skin (pelt) [13]. Therefore, it is necessary to investigate both traits measured from live animals (e.g. body weight and body length) and also the pelt. In mink, the heritability of body weight is 0.43 for females and 0.48 for males. The heritability for pelt length is 0.45 for both genders [13]. The pelt quality traits are color of the under wool (purity), length of guard hair protruding from the under wool (guard hair length), guard hair thickness, overall general impression of pelt (quality), under wool density (density) and silky appearance of the pelt (silkiness). Some of these traits also have measurement from dry skin. The heritability of this type of traits ranged from 0.06 to 0.30 [13]. Another study reported heritabilities of traits from dry skin ranging from 0.15 to 0.43 [5].

The objectives of the current study were 1) to genotype a mink breeding population using GBS to identify genome-wide set of SNPs for association with economically important traits in mink and 2) to combine association results with existing knowledge of gene function to propose candidate genes for these traits.

## Results

### SNP discovery using GBS

**Fig. 1:**
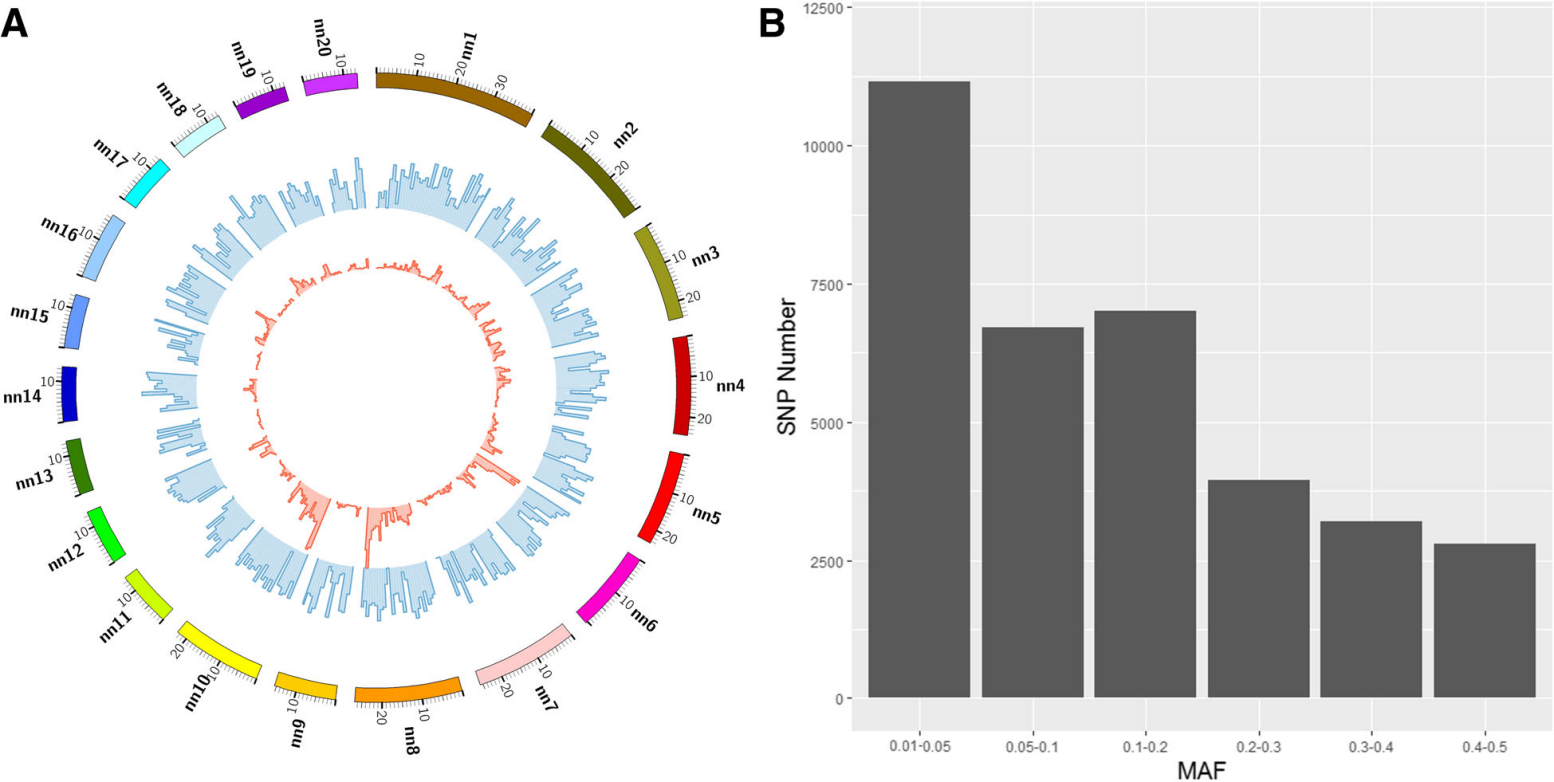
The basic statistic of SNPs called from GBS data. **a** The Circos plot shows SNP and gene densities for the first 20 scaffolds of the mink genome assembly. The ‘nn’ in the figure represents ‘*Neovison vison* scaffold’ for easy display on figure. The blue track shows the gene density in each 1 Mb block. The red track shows the SNP density of each 1 Mb block. **b** The histogram shows the number of SNP in different MAF classes

We obtained 34,816 SNPs from 2451 individuals after quality control of GBS data (Table 1). The average site quality (phred-scaled quality score) was 217,913 assuring the high quality of these selected SNPs. The number of sites for each individual and average read depth for each individual are presented in Additional file 1: Table S1. The mink genome assembly has 7175 scaffolds [9]. Of these, 945 contained at least one SNP. Among these, 335 scaffolds contained more than 10 SNPs. The distribution of SNP within and among scaffolds was uneven (Fig. 1a). Scaffold 10 had the highest number of SNPs, 1028 SNPs, followed by scaffold 8 with 1009 SNPs. As expected, longer scaffolds harbored more SNPs. Scaffolds with low SNP density were in gene-poor regions (Fig. 1a). Even though only 945 scaffolds out of 7175 had SNP markers, these scaffolds represented 93.50% of the mink genome sequence and contained 95.28% of annotated genes. The average distance between adjacent SNPs was 57 Kb with the minimal distance of 1 bp. The linkage disequilibrium (LD, r^2^) between adjacent SNPs ranged from 0.2 to 1.0. Among 21,053 identified genes, 7589 harbored at least one SNP marker in the genic region (introns and exons). In our marker set, 23,651 SNPs had minor allele frequency (MAF) > 0.05 and all SNPs had MAF > 0.01 (Fig. 1b). We kept SNPs with at least 80% calls per sample and MAF > 0.02 for further analysis. The final number of SNPs used for GWAS was 28,336.

**Table 1:** The basic SNP calling statistics using GBS data

SNP statistic	
Average site quality	217,913
Maximum distance between adjacent SNPs	5,323,641 bp
Minimum distance between adjacent SNPs	1 bp
Average distance between adjacent SNPs	57,265 bp
Scaffold number with at least one SNP	945
Scaffold number with at least two SNPs	674
Scaffold number with at least ten SNPs	335
Gene number with at least one SNP	7589
Total number of SNPs	34,816
Range of read depth	7235- 148,922
Total number of samples	2451

### Overview of association results by trait

In this study, we included twelve traits for GWAS. These were body weight, under wool density (density), guard hair length (hair length), guard hair thickness (hair thickness), color of the under wool (purity), overall general impression of pelt (quality), silky appearance of pelt (silkiness), exploratory/fearful behavior (behavior), pelt length, pelt density, pelt quality and pelt silkiness. We recorded the first eight traits on live animals, and the remaining four traits on dried skins. Table 2 provides summary statistics for these traits. In addition, we estimated the heritability and genomic heritability of each trait. Body weight and pelt length had the highest heritability and genomic heritability (Table 2).

**Table 2:** Summary statistics of the traits

Trait	Unit	Records	Mean (std. error)	Rang of trait (min~max)	Pedigree-based heritability	Genomic heritability^a^
Behavior	1 (exploratory behavior) to 4 (avoidance behavior)	875	0.22 (0.05)	−1.27~ 2.70	0.15	0.20
Body weight	gram	2265	74.59 (5.98)	− 700.97~ 868.68	0.49	0.48
Under wool density	1–3 (flat-filling)	1557	0.04 (0.01)	−1.28~ 0.85	0.10	0.15
Guard hair length	1–5 (long-short)	1557	0.03 (0.01)	−1.99~ 1.17	0.34	0.40
Guard hair thickness	1–3 (thick-thin)	1557	0.03 (0.01)	−1.4~ 1.09	0.34	0.41
Pelt density	1–3 (flat-filling)	1995	0.11 (0.01)	−1.18~ 1.20	0.22	0.21
Pelt length	cm	1960	0.06 (0.09)	−21.65~ 17.26	0.42	0.44
Pelt quality	1–12 (worst-best)	1995	0.13 (0.05)	−5.39~ 7.13	0.31	0.32
Pelt silkiness	1–3 (normal-silky)	1987	−0.08 (0.01)	−1.167~ 1.49	0.18	0.22
Color of the under wool	1–3 (red-blue)	1557	0.03 (0.02)	−1.44~ 1.22	0.19	0.13
Overall general impression of pelt	1–5 (5 is best)	1557	0.09 (0.02)	−3.33~ 1.79	0.16	0.42
Silky appearance of the pelt	1–3 (normal-silky)	1557	−0.07 (0.02)	−1.39~ 1.05	0.18	0.33

^a^ Ratio between variance captured by all SNP markers and the phenotypic variance

### GWAS for body weight

**Fig. 2:**
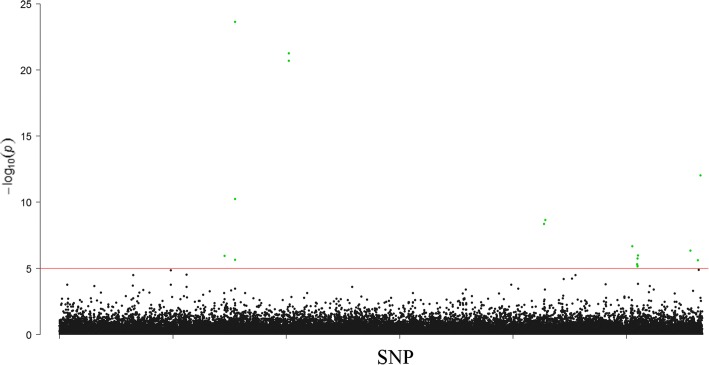
Manhattan plot for association of SNPs with body weight in mink. The red horizontal line indicates genome-wide significance level (*P* < 1.0e-5). Green dots are the genome-wide significant SNPs

The number of individuals with both genotype and phenotype records for body weight was 2352. We observed 16 SNPs showing significant association (*P* < 10^− 5^) with body weight in mink (Table 3, Fig. 2, Additional file 2: Figure S1). The most significant SNP (*P* = 2.2 × 10^− 24^) was located at 300,969 bp of scaffold43 within the *WWC3* gene. This gene belongs to the *WWC* gene family. Another closely significant SNP was located at scaffold43:154,009 (*P* = 2.3 × 10^− 6^) within the *SHROOM2* gene. This gene regulates cell morphology [14]. Two SNPs significantly associated with body weight located on scaffold70 were inside the *PPTC7* gene. The *PPTC7* gene codes for a protein phosphatase *PTC7* homolog; however, the biological link between this gene and body weight is unknown. On scaffold2192, we identified an associated SNP, but no annotated gene is in this scaffold. Another significant SNP located at scaffold205:5,542,387 (*P* = 2.2 × 10^− 9^) was located near the *MAP2K4* gene. *MAP2K4* is a key regulator of liver regeneration [15]. Phenotypes recorded in the mammalian phenotype database [16] indicate that mouse lines with mutations in this gene have lower body weight. The same scaffold has a SNP at scaffold205:760,765 (*P* = 4.2 × 10^− 9^). This SNP is located near the *WSCD1* gene. Biological function of this gene related to body weight is unknown. On scaffold326, we detected one significant SNP, scaffold326:328,684 (*P* = 2.2 × 10^− 7^). This SNP was located near the *TNKS1BP1* gene. Other positional candidate genes for the rest of the significant SNPs are *ATP2A3*, *MPRIP*, *RAB11FIP4*, *GRAP*, *EPN2*, *PEMT*, *POM121C* and *SLC5A10*, but biological links between these genes and body weight are not obvious.

**Table 3:** Genome-wide significantly associated SNPs for body weight in mink

SNP	SNP effect (gram)	*P*-value	Gene	Distance from the gene (bp)	Whether significant for pelt length
scaffold38:2633994	102.67	1.13E-6	*RAB11FIP4*	Inter-genic	T
scaffold43:300969	160.78	2.20E-24	*WWC3*	Inter-genic	T
scaffold43:154009	52.40	2.25E-6	*SHROOM2*	Inter-genic	F
scaffold70:8434089	157.92	5.34E-22	*PPTC7*	Inter-genic	F
scaffold70:8434099	155.20	2.04E-21	*PPTC7*	Inter-genic	F
scaffold205:760765	121.36	4.21E-9	*WSCD1*	15,690	T
scaffold205:5542387	112.52	2.17E-9	*MAP2K4*	445,423	T
scaffold326:328684	52.16	2.19E-7	*TNKS1BP1*	53,418	F
scaffold337:39650	87.93	4.89E-6	*EPN2*	Inter-genic	T
scaffold337:187563	98.93	1.83E-6	*GRAP*	Inter-genic	T
scaffold337:232535	93.92	7.35E-6	*SLC5A10*	Inter-genic	T
scaffold337:1044408	85.77	6.39E-6	*PEMT*	Inter-genic	T
scaffold337:1537004	93.58	1.05E-6	*MPRIP*	38,146	T
scaffold585:388001	58.39	4.64E-7	*ATP2A3*	355	F
scaffold1113:14267	96.43	2.51E-6	*POM121C*	Inter-genic	T
scaffold2192:8600	122.60	9.53E-13	NA	NA	F

Base positions refer to the mink reference genome [9]. SNP effects are given as unsigned allele substitution effects in gram

### GWAS for pelt length

**Fig. 3:**
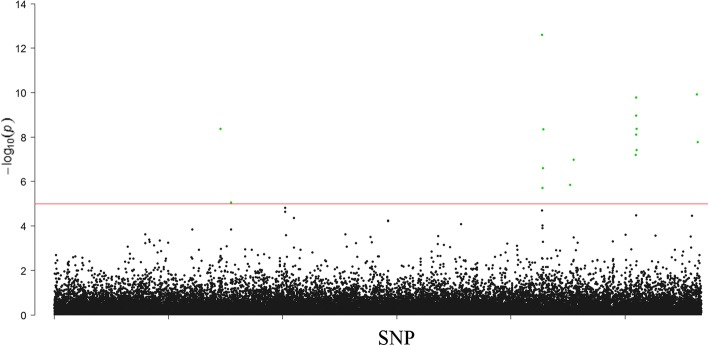
Manhattan plot for association of SNPs with pelt length in mink. The red horizontal line indicated the genome-wide significance level (*P* < 1.0e-5). The green dots indicate significantly associated SNPs

We observed 16 SNPs significantly associated with pelt length in mink (Table 4, Fig. 3, and Additional file 2: Figure S2). They were located within or close to 15 genes. Five of these genes were not shared with body weight including *USP22*, *SLC7A1*, *NDEL1*, *SMU1* and *KIF1C*. Among the 16 SNPs associated with pelt length, ten also showed significant association with body weight. Six SNPs were associated significantly with body weight, but not with pelt length. Six SNPs were associated significantly with pelt length, but not with body weight (Tables 3 and 4).

**Table 4:** Genome-wide significantly associated SNPs with pelt length in mink

SNP	SNP effect (cm)	*P*-value	Gene	Distance from the gene (bp)	Whether significant for body weight
scaffold38:2633994	1.80	4.34E-9	*RAB11FIP4*	Inter-genic	T
scaffold43:300969	−1.12	8.66E-6	*WWC3*	Inter-genic	T
scaffold205:760765	2.18	2.53E-13	*WSCD1*	15,690	T
scaffold205:1341961	0.96	1.97E-6	*KIF1C*	47,859	F
scaffold205:2221769	1.12	2.47E-07	*NDEL1*	245,367	F
scaffold205:5542387	1.64	4.53E-9	*MAP2K4*	445,423	T
scaffold245:2338413	1.33	1.40E-6	*SMU1*	50,270	F
scaffold247:347284	1.50	1.02E-7	*SLC7A1*	225,378	F
scaffold337:39650	1.53	6.37E-8	*EPN2*	Inter-genic	T
scaffold337:187563	1.95	1.65E-10	*GRAP*	Inter-genic	T
scaffold337:232535	1.85	1.08E-9	*SLC5A10*	Inter-genic	T
scaffold337:1044408	1.62	7.63E-9	*PEMT*	Inter-genic	T
scaffold337:1537004	1.54	3.87E-8	*MPRIP*	38,146	T
scaffold337:1602038	1.64	4.22E-9	*USP22*	24,238	F
scaffold1113:14267	1.94	1.22E-10	scaffold1113.1	Inter-genic	T
scaffold1256:10430	1.48	1.71E-8	NA	NA	F

Base positions are as in mink reference genome [9]

### GWAS for other traits

GWAS for other traits identified significant associations with: overall general impression of pelt, pelt quality, and pelt density (Table 5, Additional file 2: Figure S3–S5). The SNP significant for pelt density was at scaffold160:1,015,280, which was located close to the gene *ZNF148*. For pelt quality (overall general impression of pelt from the dried skin), the significant SNP was at scaffold36:12292013. This SNP is located near the gene *EBF3*. For quality (overall general impression of pelt), we found an association with an SNP at scaffold55:6477550. This SNP is located near the gene *PTPRT.* However, we have not identified a link between the functions of these genes and the phenotypes.

**Table 5:** Genome-wide significantly associated SNPs for pelt density, pelt quality and quality in mink. Base positions are as in mink reference genome [9]

SNP	Trait	SNP effect	*P*-value	Gene	Distance (bp)
scaffold36:12292013	pelt quality	0.40 (1–5 5 is best)	2.10E-6	*EBF3*	30,400
scaffold55:6477550	quality	−0.16 (1–5 5 is best)	1.62E-6	*PTPRT*	34,226
scaffold160:1015280	pelt density	0.12 (1–3, flat-filling)	4.85E-6	ZNF148	1216

## Discussion

There are several approaches to generate genome-wide markers like RFLP (restriction fragment length polymorphism), SSR and SNP. The SNP outperform other kinds of markers in terms of abundance and genotyping throughput [17]. SNP discovered from resequencing are used to facilitate population genetics [18], GWAS [19] and genomic selection [20] in livestock. For non-model species, the cost to perform population resequencing or designing a custom SNP array remains high. As a result, reduced representation sequencing including GBS provides an effective balance between information and cost [21]. In our case, the final dataset used to perform GWAS had 28,336 SNPs. However, due to the large number of scaffolds in the draft mink genome [9], we only have 945 scaffolds with at least one SNP, leaving the remaining scaffolds without any markers (Table 2). However, these 945 scaffolds contained over 93 and 95% of the genome sequence and genes respectively. Further improvements of the reference genome would help solving this problem. In addition, the improvement of the reference can also increase the accuracy and efficiency of SNP calling. More importantly, linking more scaffolds into bigger segments can help to find the genomic context of the significant SNP.

The availability of the genome assembly and genome-wide markers has made GWAS and candidate gene identification possible in mink. We performed a GWAS in mink and identified several candidate genes for four out of 12 economically important traits investigated, namely body weight, pelt length, pelt density and pelt quality. The measurement of body weight and pelt length have higher genomic heritability than the other traits (Table 2). The body weight and pelt length has heritability as 0.49 and 0.42; while the genomic heritability estimated in this study were 0.48 and 0.44, respectively. These heritability estimations were higher compare with heritabilities other traits included in this study (Table 2). Moreover, this could partially explain why we obtained better association results for these two traits. Due to the lower genomic heritability or the complexity of the genetic architecture underlying other traits, we need larger sample sizes to identify associated SNPs. Another approach would be increasing marker density and thereby increasing LD between markers and causal polymorphisms. Moreover, higher density of markers can help us to remove singleton false positive association as multiple significant SNPs in high LD to show association with the trait.

Even though our dataset is not powerful enough to identify association signals for all traits, the estimation of the genomic heritability showed that the SNPs for some traits explain sizable proportion of the phenotypic variance. The genomic heritability ranged from 0.13 (purity, color of the under wool) to 0.48 (body weight). Kenttämies & Vilva reported heritability estimates (±SE) of 0.43 ± 0.20 and 0.20 ± 0.16 for black males, and 0.07 ± 0.12 and 0.05 ± 0.12 for pastel males for general appearance in August and November, respectively [22]. Another study reported a heritability of guard hair thickness as 0.411; guard hair length as 0.153; wool density is 0.430 and quality is 0.337 [5]. Recent genetic parameters estimation of mink reported both the heritabilities on live animals and pelt. The heritabilities of traits on live animals ranged from 0.06 (wool density) to 0.48 (males body weight) [13]. The heritabilities of traits on dried skin ranged from 0.20 (pelt silkiness) to 0.45 (skin size of males and females). In our dataset, the heritability (Table 2) ranged from 0.15 (behavior) to 0.49 (body weight). Comparison of heritabilities and the genomic heritabilities for all traits indicate our marker set successfully capture most of genetics variance in most of the traits. The number of studies estimated genetic parameters for economic traits in mink is small. Comparing available results to our results showed that the markers set selected was able to capture a large proportion of genetic variances for these traits. Despite a sizable proportion of phenotypic variance was due to genetic factors, our study was unable to identify QTL for several mink traits. One reason could be that these traits are highly polygenic in nature, i.e., individual variants have small effect sizes or occur in low frequency. Furthermore, the estimation of genomic heritability showed that the marker set is suitable for genomic prediction of breeding values in mink.

GWAS has helped us to identify candidate genes for body weight and pelt length in mink. *WWC3* is a candidate gene for both body weight and pelt length. *WWC3* along with *WWC2* and *WWC1* belong to *WWC* gene family. This family was proved to be involved in Hippo signaling pathway, which is highly conserved in mammals and functions to regulate organ size [23]. *MAP2K4* is another candidate gene for body weight and pelt length in mink. This gene encodes a dual specificity mitogen-activated protein kinase 4, which acts as an essential component of the MAP kinase signal-transduction pathway. Previous research with the RNAi of *MAP2K4* showed that this gene is a key regulator of liver regeneration in mice. Mouse lines from the mammalian phenotype database [16] with mutations in this gene showed decreased body weight. *SLC7A1* is a candidate gene for pelt length. *SLC7A1* encodes the high affinity cationic amino acid transporter 1 protein. Functional studies in mice have shown that this gene plays a critical role in both hematopoiesis and growth control during mouse development. This gene is also a candidate gene for body weight, since the distance of the significant SNP (scaffold205:1,341,961) is not far apart from one of the SNPs significant for body weight (scaffold205:2,221,769). *USP22* is the ubiquitin carboxyl-terminal hydrolase 22 gene. According to uniprot [24], this gene is required for nuclear receptor-mediated transactivation and cell cycle progression. By searching the mammalian phenotype database [16], we found that mutations in this gene can cause abnormalities in many important biological process such as decreased embryo size, postnatal growth retardation, decreased number of lumbar vertebrae, embryonic lethality during organogenesis, decreased body weight and so on. We identified this gene as a candidate gene for pelt length. The significant SNP for body weight on scaffold337:1,537,004 is close to this gene. Therefore, this gene maybe considered as candidate gene.

The genome-wide markers set and QTL we presented in this study have great potential in mink breeding and genetics research. In recent years, the plant and animal breeding community have widely adopted the genomic prediction strategy [25]. As we have shown, our marker set can capture enough genomic heritability for implementing genomic prediction in mink. Moreover, with the low cost of GBS, it is possible to generate genotype data for a larger population. For the QTL we have identified there can be two impacts. On one hand, a genomic prediction model can incorporate QTL to improve the accuracy of breeding values [26]. On the other hand, comparisons of the result for similar traits from different species can help to understand the genetic architecture for the studied trait.

## Conclusion

In this study, we performed GBS in a mink population and firstly presented a genome-wide SNP makers set for mink. Subsequently, we used this genotype dataset along with twelve production traits in mink to perform the GWAS in the same population. Combining association results with existing functional information of genes and mammalian phenotype databases, we proposed *WWC3*, *MAP2K4*, *SLC7A1* and *USP22* as candidate genes for body weight and pelt length in mink.

## Methods

### Ethics approval

We used biological tissue sample during pelting of minks. Measurement of phenotypic records was performed under the routine management and breeding procedure for mink, no animal experiment and handling was involved in this study. Therefore, no ethics approval was necessary.

### Farm management

The mink were raised at the Foulum Research Farm, Aarhus University, Denmark. They were housed following Danish Legislation for mink production (Ministry of Food, Agriculture and Fisheries). The animals were either housed in two-row sheds, four-row sheds or ten-row halls, here called houses; they were exposed to natural lighting and were kept in wire cages W: 30 cm, H: 45 cm L: 90 cm connected to a wooden nest with a wire ceiling, W: 23 cm, H: 18.5 cm L: 30 cm. All cages had a wire tube cylinder, L: 32 cm, diameter: 11 cm fixed to the cage ceiling. At the age of approximately 8 weeks in July growing mink were paired off, primarily with one male and one female in each cage, and if possible often with a sibling. Minks were fed with a standard diet from a commercial company. Breeding animals were restricted in feed intake from November until flushing at the end of February. The feeding was ad lib for the rest of the year for all mink. The animals had permanent access to chopped barley straw on the nest box lid. In the breeding season, each female was only mated to one male.

### Genotyping-by-sequencing and SNP calling

We isolated DNA from toes of 2496 individuals slaughtered in Aarhus University’s mink farm, Foulum, Denmark, according to the standard protocol [27] and quantified using the Quant-iT Assay (Life Technologies). According to the protocol developed for maize [10], we generated libraries for GBS as follows. We digested one hundred ng of DNA with *PstI* and *MspI* and ligated to modified Illumina adaptors containing the restriction site overhang and unique bar-code sequence of four to nine nucleotides. Then we pooled samples to create libraries, each library consisting of up to 96 individually bar-coded DNA samples. We amplified and checked libraries for quality on an Agilent DNA 1000 Assay (Agilent Technologies, Deutschland GmbH). According to the recommendations for maize GBS libraries [10], we diluted libraries and prepared for sequencing. To generate single-end reads of 101 bp, we sequenced each library on one lane of an Illumina HiSeq4000 flow cell.

In the SNP calling procedure, initial processing was carried out on each lane separately. At first, we removed adaptor contamination using Scythe (https://github.com/vsbuffalo/scythe) with a prior contamination rate set to 0.10. Then, we used Sickle (https://github.com/najoshi/sickle) to trim reads when the average quality score in a sliding window (of 20 bp) fell below a phred score of 30. After trimming, we also discarded reads shorter than 40 bp and de-multiplexed using Sabre (https://github.com/najoshi/sabre). For alignment, we used BWA to align the reads against the mink reference genome [28]. At last, we called variants using the Genome Analysis ToolKit (GATK)’s HaplotypeCaller [29]. For variants filtering, we performed initial filtering using GATK’s SelectVariants to filter for bi-allelic sites with a mapping quality > 30. Subsequently, we used vcftools to extract variants with variant confidence/quality by depth > 3.0, Fisher Strand < 40, Strand Odds Ratio < 3.0 and variants quality/ read depth > 0.25. We also filtered the variants based on allele balance to remove loci with strong allele bias.

### Phenotypic recordings

There were three categories of traits. The first category included seven traits from the live grading in November of the first year they were born: body weight, overall general impression of pelt (quality), under wool density (density), silky appearance of the pelt (silkiness), color of the under wool (purity), length of guard hair protruding from the under wool (guard hair length), guard hair thickness. The second trait category included four traits measured on dried skins prepared for auction: pelt length, pelt quality, pelt density and pelt silkiness. The third category contains exploratory/fearful behavior (behavior), which was measured by a stick test, a voluntary approach-avoidance test used to categorize mink behavior. In the stick test, the mink was taken out from the nestbox and a test person put a tongue spatula trough the net in the front of cage. The stick test was described in details by Malmkvist and Hansen [30]. Thirstrup et al. provided a more detailed explanation of the live grading and pelt traits [13].

Animals were tested in November of the first year they were born. The behavior was scored in four categories: exploratory, avoidance, unidentified and aggressive. Only few animals were scored aggressive or unidentifiable. We excluded these two categories from the analyses. Also in November of the first year they were born, mink were graded by professional fur quality evaluators from Kopenhagen Fur (Glostrup, Denmark), after maturation of the winter fur. Minks were fixed in a tube during the live grading, and weights were registered using a handheld scale. The live animal was graded for pelt quality: 1–5 (5 is best), under wool density: 1–3 (flat-filling), silky appearance:1–3 (normal-silky), pelt clarity: 1–3 (red-blue), guard hair thickness: 1–3 (thick-thin) and guard hair length: 1–5 (long-short). Body weights of individual mink were recorded in grams. Mink were culled individually and the dried skins were prepared for auction.

Breeding males were culled after mating in the spring, breeding females were culled in November after their last litter. Progeny were culled in November in their first year. After stretching, the dried skins were scored for quality in 12 categories ranging from 1 to 12 (12 is best), skin under wool density: 1–3 (flat-filling), skin silky appearance: 1–3 (normal-silky). In addition, the skin length was measured in centimeters from the tip of the snout to tail joint.

### Phenotypes

We obtained the adjusted phenotypes (y_c_) by adjusting the original phenotypic values for fixed effects and non-genetic random effects. We analyzed these adjusted phenotypes using a linear model:

$$ {y}_c=\widehat{\mathrm{g}}+\widehat{\mathrm{e}}, $$

where $\widehat{g}$ is the estimate of the genetic effect and ê is the estimate of the random residual.

We estimated adjusting factors in a single trait BLUP animal model as follows:

$$ \boldsymbol{y}=\boldsymbol{Xb}+\boldsymbol{Wp}+\boldsymbol{Za}+\boldsymbol{e}, $$

where ***y*** is the vector of phenotypes, ***X*** is the design matrix relating fixed effects, ***b*** is the vector of fixed effects, ***W*** is the design matrix that relate litter to phenotypes, ***p*** is a vector of common litter effects, ***Z*** is the design matrix relating animals to phenotypes, ***a*** is a vector of animal effects, ***e*** is the random residual effects vector for random effects. We assumed that $\boldsymbol{p}\sim N\left(0,{I\sigma}_{pe}^2\right)$, where I is an identity matrix, ${\sigma}_{pe}^2$ is the common environment variance, $\boldsymbol{a}\sim N\left(0,A{\sigma}_g^2\right)$, where **A** is the pedigree-based relationship matrix between individuals, ${\sigma}_g^2$ is the additive genetic variance, and $\boldsymbol{e}\sim N\left(0,I{\sigma}_e^2\right)$, where I is an identity matrix, and ${\sigma}_e^2$ was the residual variance.

The fixed effects were year of birth (2006–2016), sex, and house after weaning (24 levels). In addition, we also adjusted pelt traits for age at pelting (i.e., first year or older). For behavior, there was an additional correction for the fixed effects of type of cage mate (sib/non-sib and sex of cage mate, 5 levels) and dam age (1–4). For all traits, we also adjusted for the non-genetic random effect of common litter. All records for brown mink born 2006–2016 at the research farm were included in the BLUP analyses. We recorded most traits primarily in 2013–2016. In total, there were 875 to 2265 records per trait. The pedigree file for the BLUP analysis included 85,132 mink born between 2001 and 2016. Within each trait, we scaled the adjusted phenotypes to a mean of zero, and a variance of one. We analyzed body weight, behavior, density, guard hair length, guard hair thickness, purity, quality, silkiness, pelt length, pelt density, pelt quality and pelt silkiness in this study.

We tested for significance of fixed effects in a GLM analysis in SAS (type III) (SAS version 9.3), sex was significant (*P* < 0.05) for all traits, year was not significant for quality, behavior and pelt quality. House was not significant for pelt quality and pelt density. Pelting age was not significant for pelt size. Although the fixed effects were not significant for all traits we chose to keep the same fixed effects for same category of traits for simplicity (Table 6).

**Table 6:** *P* value for fixed effect

Trait	Sex	Year	House	Pelting	Dam’sage	Cage mate
Overall general impression of pelt	0.0001	0.73	0.0003	NA	NA	NA
Under wool density	0.0001	0.0001	0.0001	NA	NA	NA
Silky appearance of the pelt	0.0007	0.0001	0.0001	NA	NA	NA
Body weight	0.0001	0.0001	0.0002	NA	NA	NA
Guard hair thickness	0.041	0.004	0.0001	NA	NA	NA
Behavior	0.0001	0.74	0.0001	NA	0.0001	0.0001
Color of the under wool	0.0001	0.0001	0.0001	NA	NA	NA
Guard hair length	0.0001	0.0001	0.001	NA	NA	NA
Pelt length	0.0001	0.0001	0.0076	0.2865	NA	NA
Pelt quality	0.0001	0.97	0.86	0.0001	NA	NA
Pelt density	0.0001	0.0001	0.21	0.0001	NA	NA
Pelt silkiness	0.0001	0.0001	0.0001	0.0001	NA	NA

### GWAS mapping and estimation of the variance explained by SNPs

Before GWAS, we removed extreme phenotype records using Tukey’s rules of quartiles ±1.5 × interquartile range. The genetic relationship matrix (GRM) was built for the mapping population by GCTA software [31]. Then we used GCTA’s GREML function to estimate the genomic heritability (variance explained by all the SNPs) for each trait. For GWAS, we used mixed model association implemented in GCTA. The *P*-value threshold for genome wide significant association was set to 10^− 5^; while the family-wise error rate at type I error of 0.05 after Bonferroni correction for 28,336 simultaneous tests was 0.05/28,336 = 1.76 × 10^− 6^. Closely located SNPs will be in LD resulting the Bonferroni multiple testing threshold being conservative. Therefore, we reported all the makers showing association at *P*-value less than 10^− 5^.

## Additional files

Additional file 1: **Table S1.** The number of sites and mean read depth for each individual of GBS population after filtering. (XLS 208 kb)

Additional file 2: **Figure S1.** The QQ-plot for body weight. **Figure S2.** The QQ-plot for pelt length. **Figure S3.** The QQ-plot for pelt density. **Figure S4.** The QQ-plot for pelt quality. **Figure S5.** The QQ-plot for quality. (DOCX 471 kb)

## Abbreviations

BAC: Bacterial artificial chromosome
GBS: Genotyping-by-sequencing
GWAS: Genome-wide association study
NGS: Next-generation sequencing
QTL: Quantitative traits loci
RAD: Restriction site associated DNA
REs: Restriction enzymes
SNP: Single nucleotide polymorphisms
