# Supplementary material for: SNP markers associated with body size and pelt length in American mink (Neovison vison)
Source: BMC Genet. 2018 Nov 12;19:103. doi: 10.1186/s12863-018-0688-6 (PMC6233529; doi:10.1186/s12863-018-0688-6)

**SNP markers associated with body size and pelt length in American mink (*Neovison vison*)**

Zexi Cai, Trine Michelle Villumsen, Torben Asp, Bernt Guldbrandtsen, Goutam Sahana, Mogens Sandø Lund

Figure S1. The QQ-plot for body weight


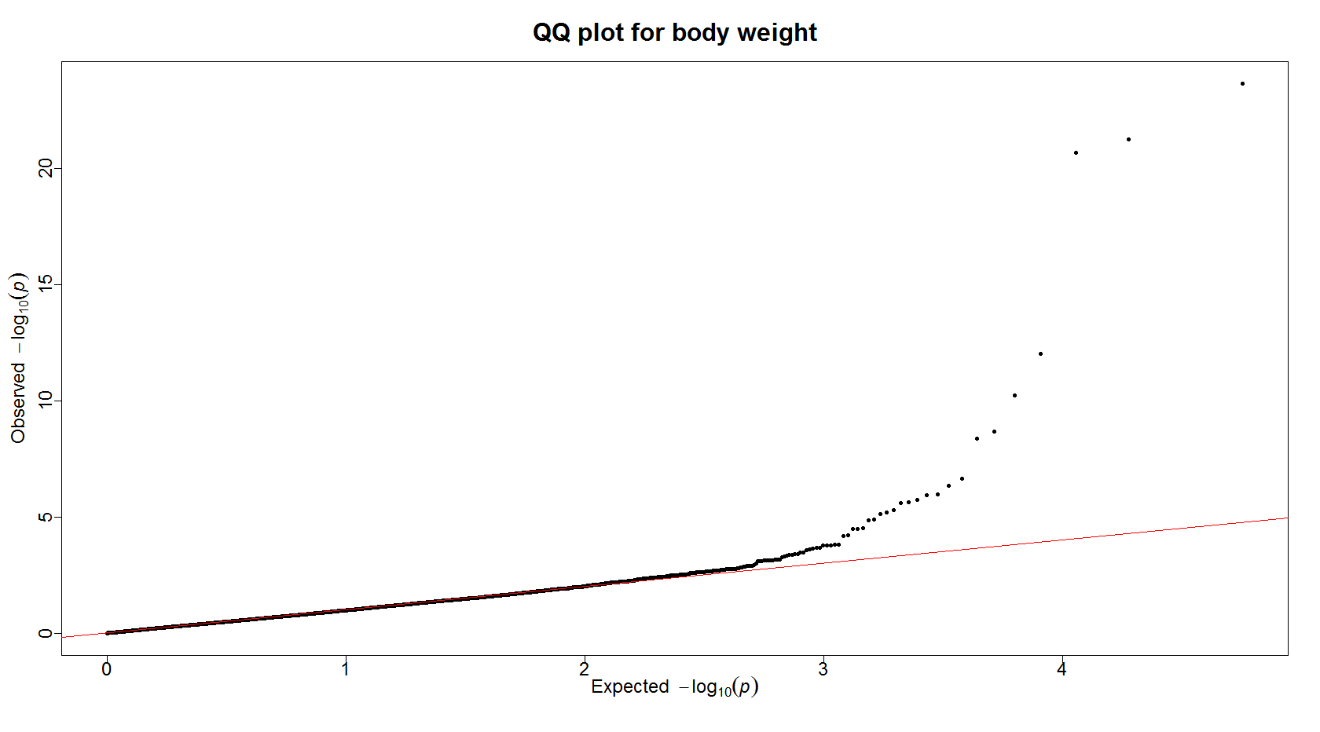


Figure S2. The QQ-plot for pelt length


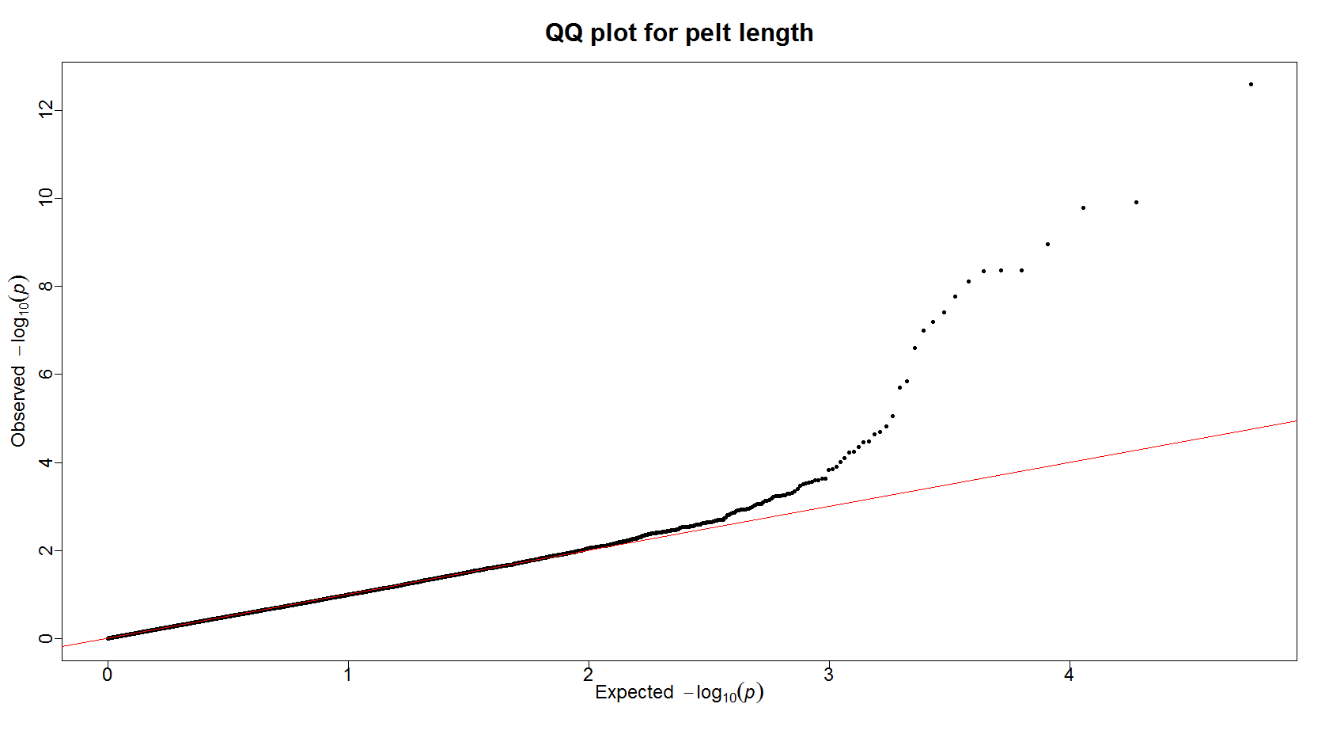


Figure S3. The QQ-plot for pelt density


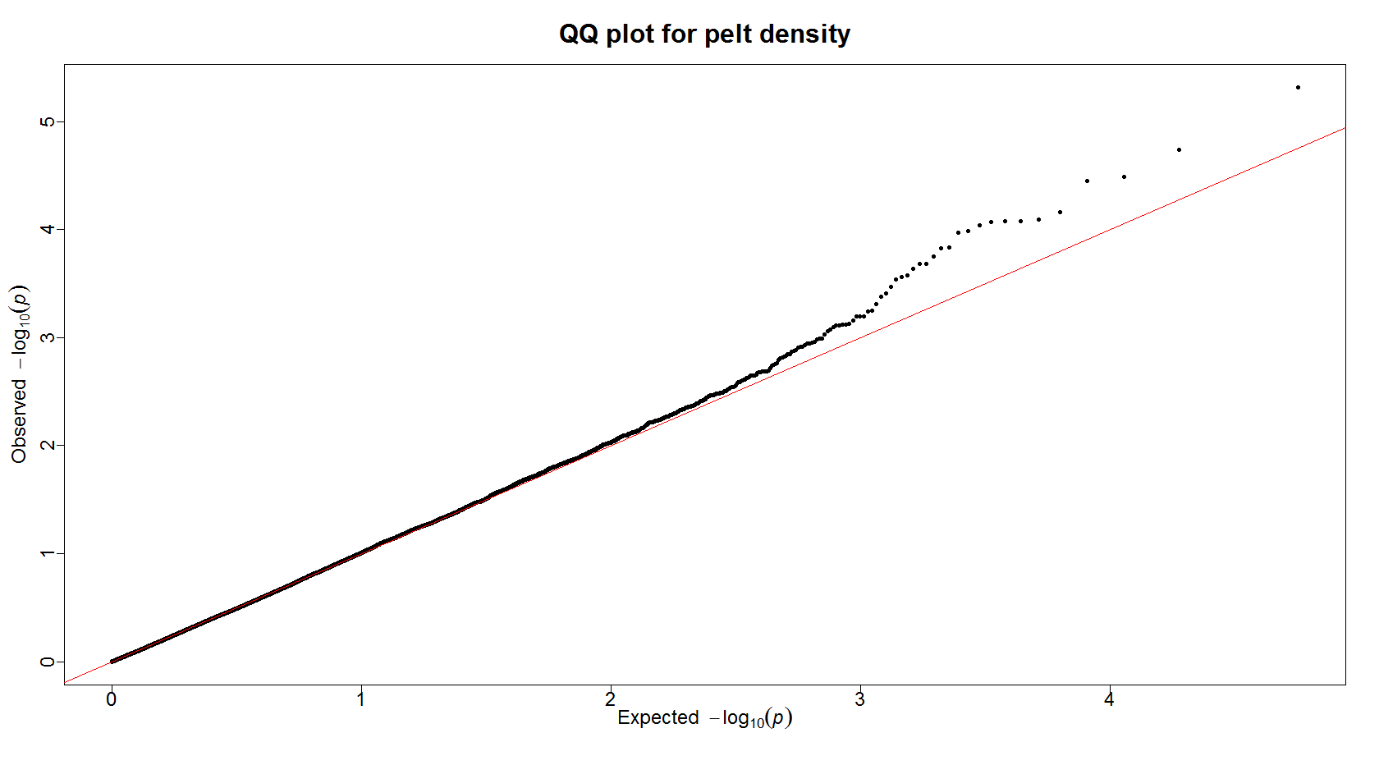


Figure S4. The QQ-plot for pelt quality


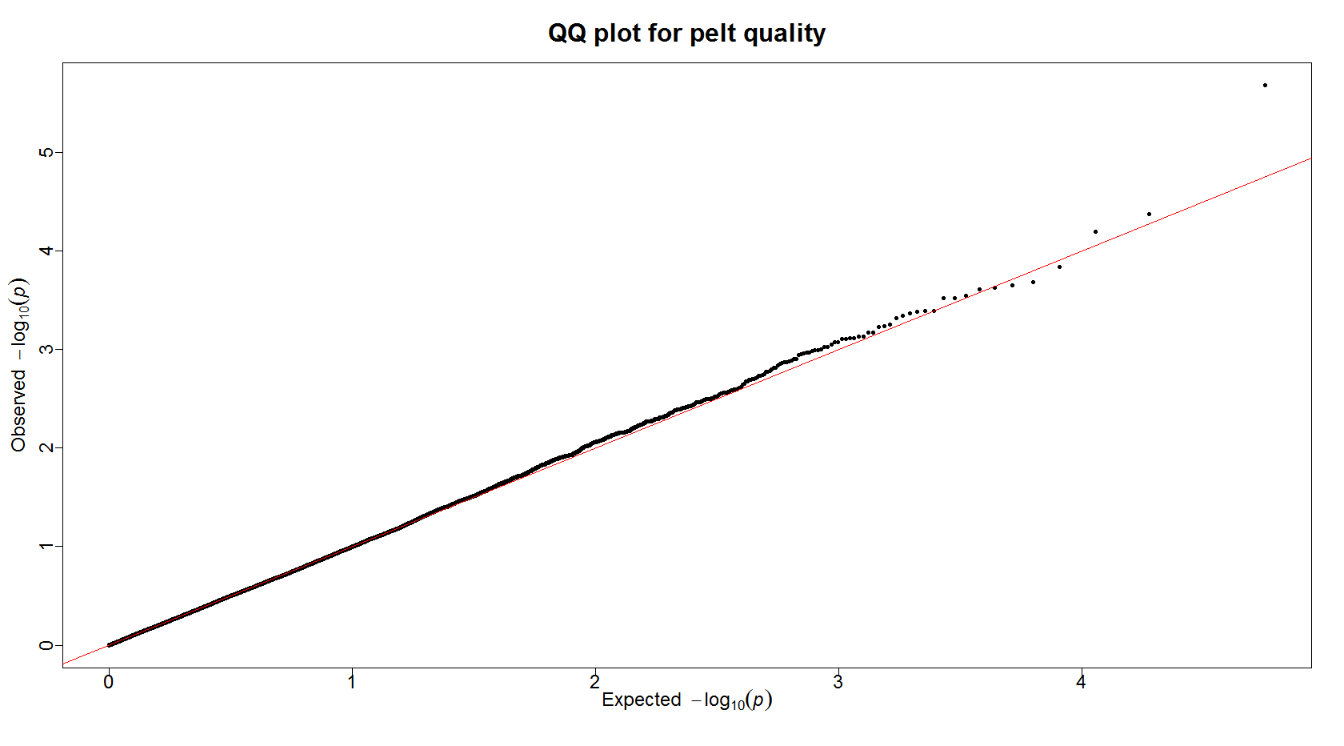


Figure S5. The QQ-plot for quality


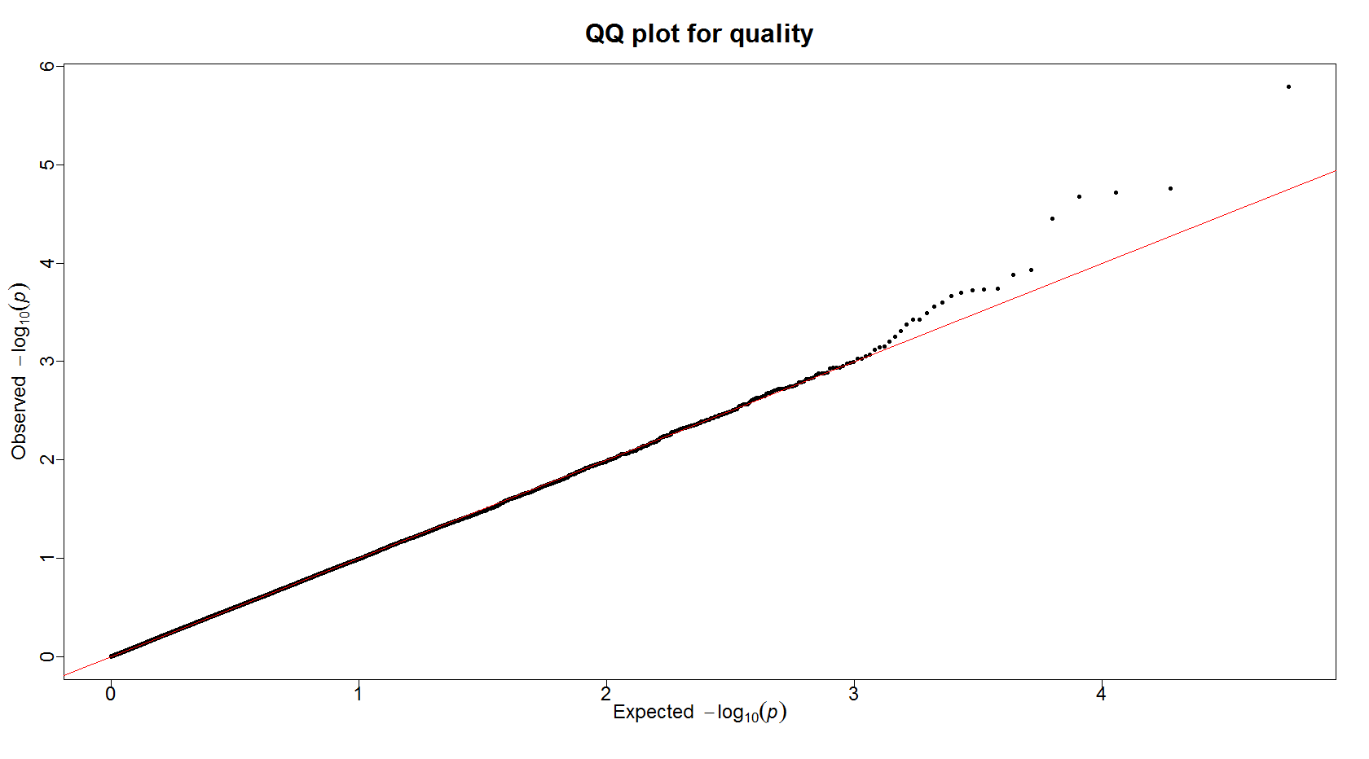

Supplement: Supplementary file 2 — Figure S1. The QQ-plot for body weight. Figure S2. The QQ-plot for pelt length. Figure S3. The QQ-plot for pelt density. Figure S4. The QQ-plot for pelt quality. Figure S5. The QQ-plot for quality. (DOCX 471 kb) [file 12863_2018_688_MOESM2_ESM.docx]
